# Supplementary material for: The genome and population genomics of allopolyploid Coffea arabica reveal the diversification history of modern coffee cultivars
Source: Nat Genet. 2024 Apr 15;56(4):721–31. doi: 10.1038/s41588-024-01695-w (PMC11018527; doi:10.1038/s41588-024-01695-w)
Supplement: Supplementary file 2 — Reporting Summary [file 41588_2024_1695_MOESM2_ESM.pdf]

nature portfolio

Corresponding author(s): Jarkko Salojärvi, Victor A. Albert, Dominique Crouzillat, Alexandre de Kochko, Patrick Descombes

Last updated by author(02/24/24

Reporting Summary

Nature Portfolio wishes to improve the reproducibility of the work that we publish. This form provides structure for consistency and transparency in reporting. For further information on Nature Portfolio policies, see our Editorial Policies and the Editorial Policy Checklist.

Statistics

For all statistical analyses, confirm that the following items are present in the figure legend, table legend, main text, or Methods section.

n/a	Confirmed	
☒	☐	The exact sample size (*n*) for each experimental group/condition, given as a discrete number and unit of measurement
☐	☒	A statement on whether measurements were taken from distinct samples or whether the same sample was measured repeatedly
☐	☒	The statistical test(s) used AND whether they are one- or two-sided<br>*Only common tests should be described solely by name; describe more complex techniques in the Methods section.*
☒	☐	A description of all covariates tested
☐	☒	A description of any assumptions or corrections, such as tests of normality and adjustment for multiple comparisons
☐	☒	A full description of the statistical parameters including central tendency (e.g. means) or other basic estimates (e.g. regression coefficient) AND variation (e.g. standard deviation) or associated estimates of uncertainty (e.g. confidence intervals)
☐	☒	For null hypothesis testing, the test statistic (e.g. *F*, *t*, *r*) with confidence intervals, effect sizes, degrees of freedom and *P* value noted<br>*Give P values as exact values whenever suitable.*
X	☐	For Bayesian analysis, information on the choice of priors and Markov chain Monte Carlo settings
☒	☐	For hierarchical and complex designs, identification of the appropriate level for tests and full reporting of outcomes
☒	☐	Estimates of effect sizes (e.g. Cohen's *d*, Pearson's *r*), indicating how they were calculated

Our web collection on statistics for biologists contains articles on many of the points above.

Software and code

Policy information about availability of computer code

Data collection	R scripts for calculating the neutral and deleterious nucletide diversities (PiNSiR) are provided in https://zenodo.org/doi/10.5281/zenodo.5136526
Data analysis	Software for genome assembly: fastQC, MHAP, Falcon, Pilon, Dovetail HiRise, SNAP, PBJelly, Trimmomatic v0.36, Lep-MAP3, samtools v1.10, Irys, HiFiasm v0.16.1. Transposable elements: REPET, fast-BLAST, MITE-Hunter, MegaBLAST,Inpactor,Sine_Finder,Repeat Masker,LTR_STRUC, Inpactor2,DensityMap,tRNAscan-SE 2.0,RNAmmer,snoStrip, INFERNAL v1.1.2,cmsearch. RNAseq: AdapterRemoval,HISAT2 v2.2.0, StringTie v2.1.2, gffread v0.12.1, RNAplonc v1.1, BEDTools v2.26.0, Quality control and annotation: quast, BUSCO, webApollo, Portcullis, Mikado, Augustus, Genmark ,SNAP, Maker. Data analysis: CoGe SynMap, R, BWA mem v0.7.16a-r1181, picard v2.18.14, GATK v3.8.0, BWA samse, MapDamage v.2.0.8, snpEff v4.3t, Cutadapt v2.10, VCFtools v.0.1.17, ANGSD v.0.933, Plink v1.90, ADMIXTURE, RAxML, PSMC, bcftools, SMC++, KING v2.2.5, Admixtools, Orientagraph v1.0, Fastsimcoal v. 2.6.0.3, TIP_finder, Adegenet v. 2.1.3.

For manuscripts utilizing custom algorithms or software that are central to the research but not yet described in published literature, software must be made available to editors and reviewers. We strongly encourage code deposition in a community repository (e.g. GitHub). See the Nature Portfolio guidelines for submitting code & software for further information.

Data

Policy information about availability of data

All manuscripts must include a data availability statement. This statement should provide the following information, where applicable:

- Accession codes, unique identifiers, or web links for publicly available datasets
- A description of any restrictions on data availability
- For clinical datasets or third party data, please ensure that the statement adheres to our policy

Coffee genome assemblies are available at CoGe (https://genomevolution.org/): C. canephora: 50947, C. eugenioides: 67315, and C. arabica: 66663 (Pacbio HiFi) and 53628 (Pacbio). The genome data is also available at ORCAE (https://bioinformatics.psb.ugent.be/orcae/overview/Coara and https://bioinformatics.psb.ugent.be/gdb/coffea_arabica/). All sequencing data are available at NCBI under bioproject ID PRJNA698600, and our assemblies are accessioned there as JAZHSI000000000.1, JAZHGF000000000.1, JAZHGH000000000.1, and JAZHGG000000000.1. Genotyping data (VCF files) and syntenic alignments are available in Data Dryad: https://doi.org/10.5061/dryad.qnk98sfpt .

Field-specific reporting

Please select the one below that is the best fit for your research. If you are not sure, read the appropriate sections before making your selection.

☐ Life sciences ☐ Behavioural & social sciences ☒ Ecological, evolutionary & environmental sciences

For a reference copy of the document with all sections, see nature.com/documents/nr-reporting-summary-flat.pdf

Ecological, evolutionary & environmental sciences study design

All studies must disclose on these points even when the disclosure is negative.

Study description	We carried out reference genome sequencing, assemblies and genome annotations of Coffea arabica ET-39 di-haploid, C. canephora DH 200-94, and C. eugenioides Bu-A accessions. This resubmission includes a new and high-quality PacBio Hifi-based assembly. Assembly was followed by a study of the genome evolution of the tetraploid C. arabica, analysis of expression dominance in specific biochemically important pathways. Next we studied the population history of C. arabica using 39 whole-genome sequenced accessions including wild and cultivated representatives. Finally, we analysed individuals containing recent introgression from C. canephora.
Research sample	A population of wild C. arabica representatives collected from different locations around Ethiopia during the 1960's, representing a large proportion of the geographic range of extant wild populations, was employed. Representatives of the two most commercially important C. arabica cultivar lines, Typica and Bourbon, and their crosses, were also examined. Additionally, the relatively new cultivar Geisha, which has recently become commercially important, and the lectotype individual of C. arabica from the Linnaean Society, dating back to 1700s, were sampled. Five lines were used that descended from a spontaneous C. canephora x C. arabica hybrid identified in Timor.
Sampling strategy	Leaves were collected from the selected individuals and sequenced to high coverage using Illumina short-read sequencing. The samples were chosen based on accessibility to material. For the cultivar lines Bourbon and Typica, the sample size was sufficient since both lines date back to a single plant bottleneck in the 1700s. Wild representatives were collected during two missions to Ethiopia in the 1960s, and they represent the diversity of wild C. arabica plants; this was verified by comparing the sequenced individuals to a wider collection where marker-based analysis had already been carried out and published.
Data collection	Wild representatives were collected during two missions to Ethiopia in 1960s by FAO and IRD. Cultivars were obtained from plant breeding experts in IRD, IAPAR, EMBRAPA, Nestle, ICCRI, IAC - Campinas, and NARO, as well as from conservation Institutes Amsterdam Botanical Garden and Natural History Museum London.
Timing and spatial scale	Wild representatives were collected in the 1960's in Ethiopia.
Data exclusions	No exclusion of data.
Reproducibility	No biological experiments were carried out in the work. All statistical analyses report a p-value associated with the analyses aimed to assess the reproducibility of the results.
Randomization	No clinical experimentation was done in the paper, therefore there was no need for randomisation.
Blinding	Blinding was not possible since the data analysis did not contain case vs. control experimental setups.

Did the study involve field work? ☐ Yes ☒ No

Reporting for specific materials, systems and methods

We require information from authors about some types of materials, experimental systems and methods used in many studies. Here, indicate whether each material, system or method listed is relevant to your study. If you are not sure if a list item applies to your research, read the appropriate section before selecting a response.

Materials & experimental systems

n/a	Involved in the study	
☒	☐	Antibodies
☒	☐	Eukaryotic cell lines
☒	☐	Palaeontology and archaeology
☐	☒	Animals and other organisms
☒	☐	Human research participants
☒	☐	Clinical data
☒	☐	Dual use research of concern

Methods

n/a	Involved in the study	
☒	☐	ChIP-seq
☒	☐	Flow cytometry
☒	☐	MRI-based neuroimaging

Animals and other organisms

Policy information about studies involving animals; ARRIVE guidelines recommended for reporting animal research

Laboratory animals	*For laboratory animals, report species, strain, sex and age OR state that the study did not involve laboratory animals.*
Wild animals	*Provide details on animals observed in or captured in the field; report species, sex and age where possible. Describe how animals were caught and transported and what happened to captive animals after the study (if killed, explain why and describe method; if released, say where and when) OR state that the study did not involve wild animals.*
Field-collected samples	Wild representative samples were collected from Ethiopia in 1960s during two missions, and since then have been maintained in the field in different locations in Ecuador, Reunion (France), Brasil and Ethiopia.
Ethics oversight	*Identify the organization(s) that approved or provided guidance on the study protocol, OR state that no ethical approval or guidance was required and explain why not.*

Note that full information on the approval of the study protocol must also be provided in the manuscript.
